# Diagnostic and Prognostic Performance of Metabolic Signatures in Pancreatic Ductal Adenocarcinoma: The Clinical Application of Quantitative NextGen Mass Spectrometry

**DOI:** 10.3390/metabo14030148

**Published:** 2024-02-29

**Authors:** Paulo D’Amora, Ismael D. C. G. Silva, Steven S. Evans, Adam J. Nagourney, Katharine A. Kirby, Brett Herrmann, Daniela Cavalheiro, Federico R. Francisco, Paula J. Bernard, Robert A. Nagourney

**Affiliations:** 1 Metabolomycs, Inc., 750 E. 29th Street, Long Beach, CA 90806, USA; pdamora@metabolomycs.com (P.D.); idale@metabolomycs.com (I.D.C.G.S.); sevans@nagourneyci.com (S.S.E.); pbernard@nagourneyci.com (P.J.B.); 2 Nagourney Cancer Institute, 750 E. 29th Street, Long Beach, CA 90806, USA; anagourney@nagourneyci.com (A.J.N.); bherrmann@nagourneyci.com (B.H.); dcavalheiro@nagourneyci.com (D.C.); efrancisco@nagourneyci.com (F.R.F.); 3 Gynecology Department, School of Medicine of the Federal University of São Paulo (EPM-UNIFESP), Rua Pedro de Toledo 781—4th Floor, São Paulo 04039-032, SP, Brazil; 4 Center for Statistical Consulting, Department of Statistics, University of California Irvine, (UC Irvine), 843 Health Science Rd., Irvine, CA 92697, USA; kathark@uci.edu; 5 Department of Obstetrics and Gynecology, University of California Irvine (UC Irvine), 101 The City Dr S, Orange, CA 92868, USA

**Keywords:** pancreatic cancer, NextGen metabolomics, metabolic profiling, biomarker, early detection, survival analysis, prognostication

## Abstract

With 64,050 new diagnoses and 50,550 deaths in the US in 2023, pancreatic ductal adenocarcinoma (PDAC) is among the most lethal of all human malignancies. Early detection and improved prognostication remain critical unmet needs. We applied next-generation metabolomics, using quantitative tandem mass spectrometry on plasma, to develop biochemical signatures that identify PDAC. We first compared plasma from 10 PDAC patients to 169 samples from healthy controls. Using metabolomic algorithms and machine learning, we identified ratios that incorporate amino acids, biogenic amines, lysophosphatidylcholines, phosphatidylcholines and acylcarnitines that distinguished PDAC from normal controls. A confirmatory analysis then applied the algorithms to 30 PDACs compared with 60 age- and sex-matched controls. Metabolic signatures were then analyzed to compare survival, measured in months, from date of diagnosis to date of death that identified metabolite ratios that stratified PDACs into distinct survival groups. The results suggest that metabolic signatures could provide PDAC diagnoses earlier than tumor markers or radiographic measures and offer insights into disease severity that could allow more judicious use of therapy by stratifying patients into metabolic-risk subgroups.

## 1. Introduction

The global burden of pancreatic cancer (pancreatic ductal adenocarcinoma—PDAC) continues to rise, with the incidence predicted to increase 1.97-fold by 2060 [1]. In the US, pancreatic cancer represents only 3.3% of newly diagnosed malignancies but is responsible for 8.3% of annual cancer deaths. Only 1–2% of patients with advanced disease survive 3 years [2], yet advanced disease represents the majority of newly diagnosed patients [3]. Earlier diagnosis and better prognostication offer the opportunity to treat this disease while it remains curable. As our understanding of this disease at a molecular level continues to improve, insights gained have not translated into better outcomes, as the principal treatments for advanced disease have remained unchanged for over a decade. Genomic approaches for early detection have rapidly advanced but continue to suffer from false positives and false negatives [4,5]. One commercially available test revealed a sensitivity of only 16.8% [6] in Stage I solid tumors, yet detection at this stage offers the best and possibly only chance of curative intervention. More importantly, next-generation sequencing (NGS)-based genomic platforms cannot offer insights into disease severity and prognosis [7].

The growing recognition that cancers utilize metabolic re-programming to gain survival and metastatic advantage has led to a renewed interest in the study of cancer metabolism [8].

Epidemiologic studies have identified obesity and Type 2 Diabetes Mellitus as risk factors for pancreatic cancer [9]. This was the subject of a subsequent review [10], and *KRAS* mutation, highly prevalent in PDAC, has been strongly associated with metabolic re-programming as a driver of pancreatic tumorigenesis [11]. Alterations in lipid metabolism are also features of malignant transformation [12], with a recent report showing that *KRAS* G12D mutation, one of the more common variants in PDAC, drives tumorigenesis through the upregulation of *SLC25A1* [13].

As the tools to study human metabolism continue to improve, one of the most important advances has been the application of targeted mass spectrometry using tandem MS/MS to quantify micro-molar concentrations of metabolites in the blood and other body fluids. Targeted metabolomics has allowed the development of metabolite ratios that serve as highly reproducible metrics of malignant transformation. Using algorithms and machine learning, investigators have identified tumor-specific metabolite signatures that can identify pancreatic cancer and provide prognostic information for survival [14].

The application of targeted metabolomics to pancreatic cancer diagnostics remains in its infancy, but several groups have made important strides mostly through the application of un-targeted MS/MS [15].

By applying targeted MS/MS, we have been able to reproducibly measure concentrations of critical metabolites to craft unique clinical signatures that now allow us to move the field from scientific exploration to clinical application [16].

The hypothesis to be tested was that pancreatic cancer patients manifest metabolic changes, reflected by alterations in amino acids, lipids, acylcarnitines and biogenic amines that could be measured in the plasma and serve as diagnostic tests for the early detection of disease. We then explored whether these and related pancreatic adenocarcinoma metabolic signatures could offer insights into disease prognosis and survival measured from time of diagnosis to time of death.

As we have shown in breast [17] and ovarian [18] cancers, comprehensive profiling of cancer-related metabolic alterations offers diagnostic, prognostic and predictive insights that can inform clinical therapy decisions. As genotypes translate into phenotypes through intricate metabolic networks, metabolomics has the potential to offer a powerful tool to interrogate the complexity of pancreatic cancer biology [19,20,21,22].

This study examines the critical role of targeted metabolomics in the diagnosis and prognosis of pancreatic cancer and underscores the potential of quantitative metabolomics for the study of pancreatic cancer biology.

## 2. Materials and Methods

### 2.1. Study Design and Patient Accrual

This study was conducted at the Nagourney Cancer Institute and Metabolomycs, Inc., located in Long Beach, CA, USA. The protocol was approved after first revision on 31 October 2016 by the Western IRB (WCG/Conexus approval #20162430, confirmation ID #45068204) and was conducted in compliance with the World Medical Association Declaration of Helsinki. Written informed consent was obtained from all participants.

All patients who were accrued for this study (n = 30) had pathologic confirmation of pancreatic ductal adenocarcinoma. Clinical characteristics of the PDAC patients are provided in Supplemental Table S1. The control group (n = 60) was composed of healthy volunteers. Volunteers submitted plasma samples as part of an ongoing IRB-approved protocol designed to examine metabolic profiles in otherwise healthy normal individuals. Control group clinical characteristics are included in Supplemental Table S2. All patients and controls submitted an ethylenediaminetetraacetic acid (EDTA)-purple-top tube collected from peripheral blood samples, obtained at the time of protocol accrual. All patients and control subjects provided written informed consent for participation in the study protocol.

### 2.2. Inclusion and Exclusion Criteria

Between 30 November 2016 and 20 April 2023, 30 patients with histologically confirmed pancreatic ductal adenocarcinoma and 60 healthy volunteers submitted 5 mL of EDTA-anticoagulated blood. All patients fasted for 12 h prior to phlebotomy.

### 2.3. Clinical and Laboratory Data Assessment

Clinical data were extracted by chart review from physician notes and medical records in the documents provided to the accruing physician obtained at the time of clinical consultation.

### 2.4. Study Outcomes

The primary endpoint was overall survival measured in months, from the date of diagnosis to date of death or last follow-up.

### 2.5. Collection of Blood Samples

Peripheral venous blood samples from each patient/volunteer were collected using tubes with anti-clotting factor (EDTA). Immediately after blood collection, samples were centrifuged (5 min at 4000 rpm). After centrifugation, the plasma was aliquoted, frozen and stored at −80 °C for targeted mass spectrometry analysis.

### 2.6. Metabolomic Analysis Workflow

**Figure 1:**
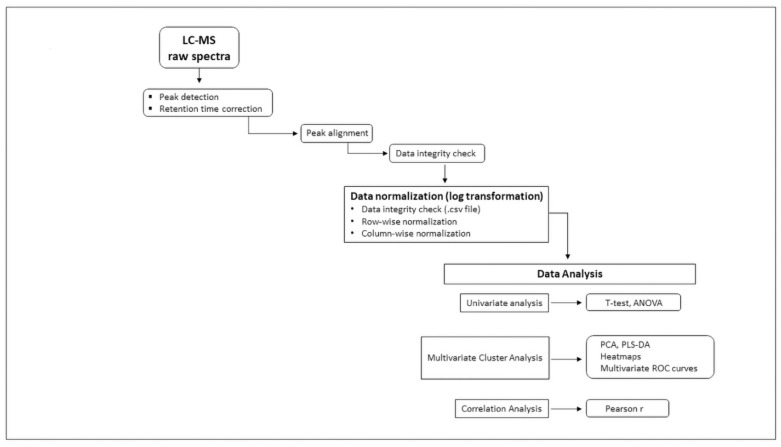
Flowchart illustrating workflow and data processing—individual metabolite absolute concentrations measured by targeted mass spectrometry (MS/MS) transmitted in .csv format data files were log-transformed for normalization and then uploaded into MetaboAnalyst 5.0 bio-informatic data analytic platform. Univariate (*t*-test, ANOVA), multivariate Principal Component Analysis (PCA), Partial-Least-Squares Discriminant Analysis (PLS-DA), Heat maps, multivariate Receiver Operating Characteristic (ROC) analysis and correlation coefficients (Pearson r) were applied to identify metabolites and ratios associated with pancreatic ductal adenocarcinoma.

Figure 1 provides sample processing flowchart from plasma receipt to liquid chromatography and mass spectrometry through data analysis.

### 2.7. Pre-Analytic Sample Processing

All subjects were accrued on site, both PDAC and controls, and fasted for 12 h prior to accrual. Plasma sample preparation was carried out according to manufacturer’s protocol. The AbsoluteIDQ^®^ p180 kit, Biocrates AG Life Sciences (Innsbruck, Austria) is an automated platform that utilizes phenyl isothiocyanate (PITC) derivatization of the target analytes in bodily fluids with internal standards for quantitation. Amino acids and biogenic amines were determined by LC-MS mode. Acylcarnitines, phospholipids (lysophosphatidylcholines, phosphatidylcholines, sphingomyelins) and hexoses were analyzed in flow injection analysis (FIA) mode. Briefly, 10 µL of human plasma was transferred to the 96-well plate and dried under a nitrogen stream. Then, 50 µL of a 5% PITC solution was added to derivatize amino acids and biogenic amines. After incubation, the filter spots were dried before metabolites were extracted using 5 mM ammonium acetate in methanol (300 µL). These were transferred into the 96-well plate for analysis after further dilution using the MS running solvent A (formic acid solution). Quantification was carried out using internal standards and a calibration curve [23].

Each p180 plate contained 16 controls including quality control samples (QC) and internal standards. External validation was provided using the National Institute of Standards and Technology (NIST) human frozen plasma standards [24]. Samples were repeated at 3, 6 and 12 months to ensure consistency over time to control for any batch effect.

### 2.8. Targeted Quantitative MS/MS Analysis

Targeted metabolomics using annotated metabolites is a comparatively new discipline. In summary, data generated by liquid chromatography coupled with tandem mass spectrometry (LC-MS/MS) provides micro-molar (mMolar) concentrations that are uploaded into MetaboAnalyst platform. MetaboAnalyst compares concentrations against their database, identifying metabolites of interest as those that fall above or below normal ranges. The MetaboAnalyst platform is designed to apply unsupervised and supervised clustering analyses that identify the metabolites and ratios that are the most discriminating between groups.

In this study, targeted metabolomic analyses of plasma samples were performed using Absolute IDQ^®^ p180 kit from the company Biocrates Life Science AG (Innsbruck, Austria). This validated targeted assay allows simultaneous detection and absolute quantification of metabolites in plasma in a high-throughput manner. This kit can be used on a variety of LC-MS/MS instruments and has already been applied to many studies of human serum and plasma, including several large-scale prospective cohort studies [24]. Absolute quantification (μMolar) of blood metabolites was achieved by targeted quantitative profiling of 186 annotated metabolites by electrospray ionization (ESI) tandem mass spectrometry (MS/MS) in plasma samples, blinded to any phenotype information, on a SCIEX Citrine™ LC-MS/MS 6500+ (MD) mass spectrometry platform. Briefly, a targeted profiling scheme was used to quantitatively screen for fully annotated metabolites using multiple reaction monitoring, neutral loss and precursor ion scans. Quantification of metabolite concentrations and quality control assessment were performed with the MetIDQ^TM^ software package (Biocrates Life Sciences AG, Innsbruck, Austria), which implies proof of reproducibility within a given error range. An MS-excel file (.csv) was then generated, which contained sample identification and 186 metabolite names and concentrations measured in µMolar. MetaboAnalyst is a web-based server capable of analyzing both chemo-metric and quantitative data sets using analytic tools borrowed from micro-array analyses. For quantitative (targeted) studies, compound concentration tables are applied.

Several computational tools are utilized, including probabilistic PCA (PPCA) and Bayesian PCA (BPCA) followed by data normalization using four principal modalities. This is then submitted to a series of well-established statistical analyses, including univariate analyses (fold-change, *t*-test and volcano plot).

PCA is an unsupervised platform. PLS-DA is a supervised method that uses multiple linear regression applications. Variable importance plots (VIPs) are used to select the most important features in the data. Heat maps provide the opportunity for clustering analyses and self-organizing maps (SOMs).

Applying the metabolites to the Human Metabolome Database (HMDB) provides the opportunity for pathway mapping to place the results in the context of known cellular biochemical reactions.

For metabolomic data analysis, log transformation was applied to all quantified metabolites to normalize the concentration distributions and then uploaded into the web-based analytical pipeline’s MetaboAnalyst 5.0 [25] and Receiver Operating Characteristic Curve Explorer and Tester (ROCCET) for the generation of uni- and multivariate Receiver Operating Characteristic (ROC) curves obtained through Support Vector Machine (SVM), Partial-Least-Squares Discriminant Analysis (PLS-DA). Random Forests as well as logistic regression models were used to calculate odds ratios of specific metabolites. ROC curves were generated by Monte Carlo Cross-Validation (MCCV) using balanced sub-sampling where two thirds (2/3) of the samples were used to evaluate the feature importance. Significant features were then used to build classification models, which were validated on 1/3 of the samples that were left out of the first analysis. The same procedure was repeated 10–100 times to calculate the performance and confidence interval of each model. To further validate the statistical significance of each model, ROC calculations included bootstrap 95% confidence intervals for the desired model specificity as well as accuracy after 1000 permutations and false discovery rate (FDR) calculations.

### 2.9. Metabolite Panel

In total, 186 annotated metabolites were quantified using the Biocrates AbsoluteIDQ^®^ p180 kit (Biocrates Life Sciences AG, Innsbruck, Austria): LC-MS/MS (42 small molecules): 21 amino acids, 21 biogenic amines; FIA-MS/MS (145 Lipids): 40 acylcarnitines, 90 glycerophospholipids, 15 sphingomyelins and a sum of monosaccharides. Supplemental Table S3 provides a comprehensive list of all of the analytes included in the p180 kit, together with the standard nomenclatures.

### 2.10. Statistical Analysis

Sample characteristics were evaluated with continuous variables expressed as means and standard deviation and categorical variables as frequencies and percentages. Logistic regression models were fit to compare the effects of each metabolite measure as a potential predictor on each clinical outcome, both with and without control for age and sex to estimate unadjusted and adjusted odds ratios (ORs) and 95% confidence intervals (CIs) for the association between each metabolite and each outcome. p-Statistical significance was set at *p* < 0.05. Statistical analyses for these clinical outcome models were performed using R version 4.0.1.

### 2.11. Diagnostic Analysis

We initially conducted an exploratory analysis using targeted MS/MS in plasma samples from 10 patients with histologically confirmed PDAC and compared the results from a collection of 169 plasma samples comprising healthy controls. An additional analysis was conducted comparing the 10 PDAC patient plasma samples with a collection of other cancers and non-malignant conditions (Supplemental Figure S8).

### 2.12. Prognostic Analysis

To assess the prognostic impact of the PDAC metabolic signatures, we compared the numeric values of the metabolic ratios to patient survival measured in months, from the date of diagnosis to date of death: range 3–159 months, median of 15 months, mean of 25.8 months. Applying machine learning, the most discriminating metabolite ratios were identified in PDAC patient plasma that were capable of segregating patient survivals in months (total of PDAC n = 30).

### 2.13. Survival Analysis

Data from 30 pancreatic cancer patients, 4 of whom were alive at the end of follow-up, had C4/C4:1 ratio categorized into 2 equally sized groups based upon whether the ratio fell above or below the median value, 6.87. Kaplan–Meier curves were used to plot survival probabilities in the two groups. Cox proportional hazards regression was used to assess the association between a C4/C4:1 ratio falling above or below the median value with the hazard of death. We assessed the proportional hazards assumption using the zero-slope test and log–log plots and found that it was not violated. Statistical analyses were performed using StataCorp. 2023. Stata Statistical Software: Release 18 (StataCorp LLC, College Station, TX, USA).

## 3. Results

**Figure 2:**
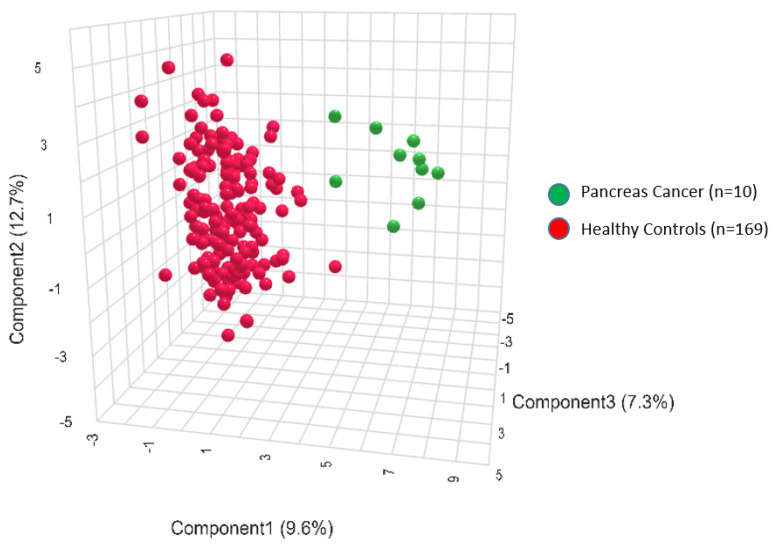
Three-dimensional projection of the metabolite profiles in the 2 groups: pancreatic cancer (green) and controls (red). PLS-DA is a supervised multivariate analysis, clustering pancreatic cancer patient (green) and control groups (red) according to their metabolic profiles.

An exploratory analysis was conducted using quantitative mass spectrometry to examine metabolic signatures associated with PDAC. A cohort of healthy controls provided a comparator for the PDAC-associated metabolic profiles. As can be seen in Figure 2, the PLS-DA analysis conducted using all 186 metabolites from the p180 kit clearly separated the PDAC group from the controls (Figure 2).

**Figure 3:**
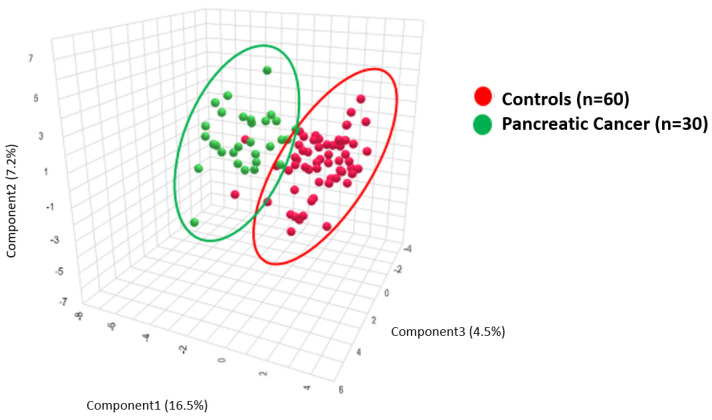
PLS-DA clustering analysis demonstrates biochemical differences between the plasma of pancreatic cancer patients (green) and plasma from healthy controls (red).

To validate these findings, we conducted a confirmatory PLS-DA analysis that compared PDAC patient metabolic profiles, n = 30 (median age = 65.5 years, range 41–85 years, 15/30 (50%) female, 15/30 (50%) male), with age- and sex-matched controls, n = 60 (median age 66, range 57–92, 33/60 (55%) female, 27/60 (45%) male (Supplemental Tables S1 and S2)). Results are provided in Figure 3.

Figure 3 provides the PLS-DA of the metabolite ratios that compare 30 PDAC plasma samples to 60 age- and sex-matched healthy controls.

**Figure 4:**
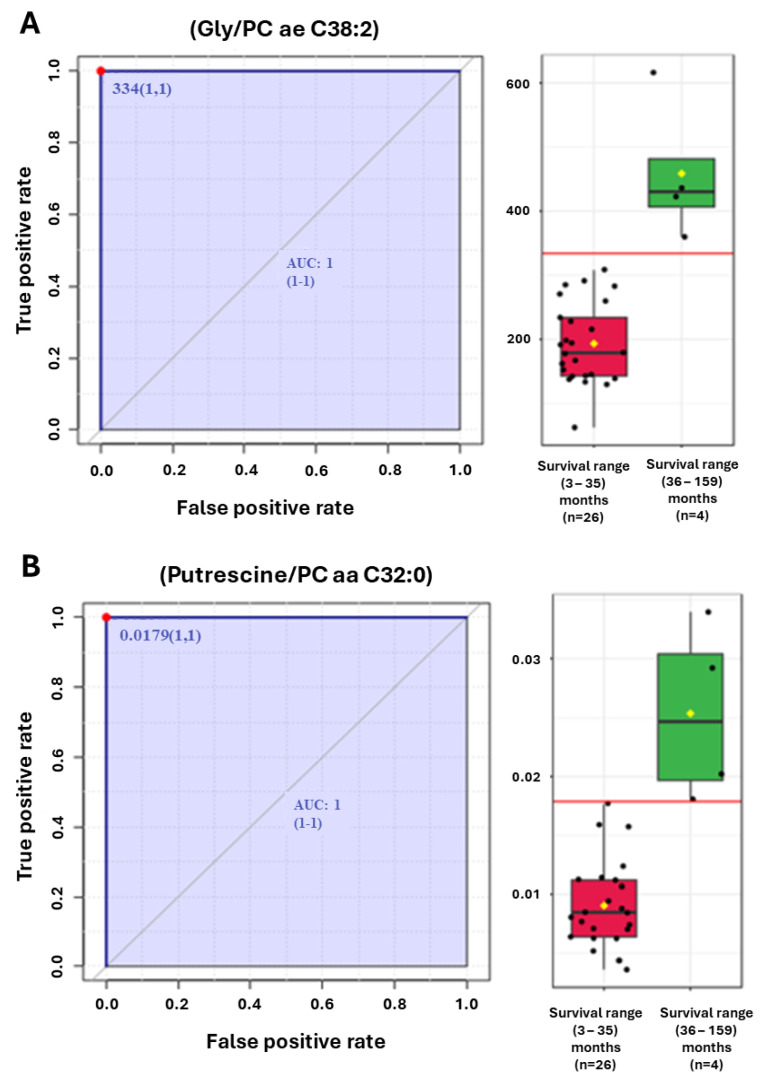
(**A**,**B**) ROC analyses of (**A**) Glycine/phosphatidylcholine (PC) ae C38:2 and (**B**) Putrescine/PC aa C32:0 ratios that segregate patients with the longest overall survival.

To assess the impact of metabolic signatures upon survival, we compared metabolite ratios with overall survival, measured in months from the date of diagnosis to the date of death or last follow-up. Figure 4A,B provide examples of two of the most discriminating ratios for PDAC patient survival (Gly/PC ae 38:2) and (Putrescine/PC aa 32:0) as ROC analysis (Figure 4).

**Figure 5:**
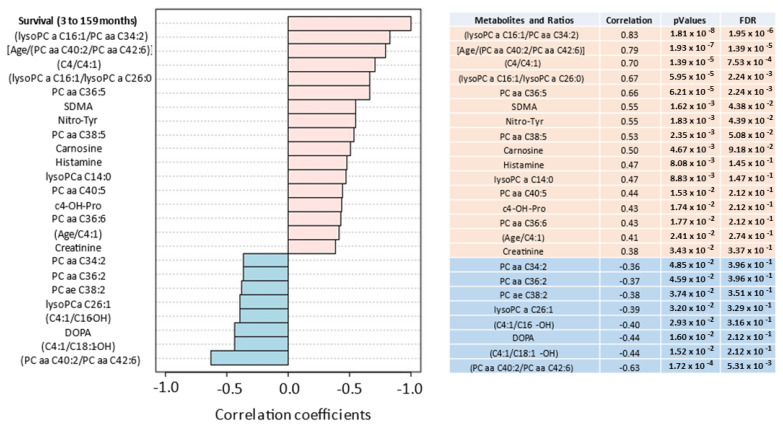
Pearson Moment (R-values) correlates the most discriminating metabolites and ratios with patient survival. Positive (salmon) and negative (teal) correlations are provided from 30 pancreatic cancer patients with a median overall survival of 15 months, mean of 25.8 months and range of 3 to 159 months.

Pearson Moment correlation coefficients were examined that correlated metabolites and metabolite ratios with PDAC survival (median of 15 months, mean of 25.8 months, range 3–159 months), as provided in Figure 5. For comparison, we provide the same metabolite and ratio analysis for Pearson Moment correlations with PDAC diagnosis (Supplemental Figure S9).

**Figure 6:**
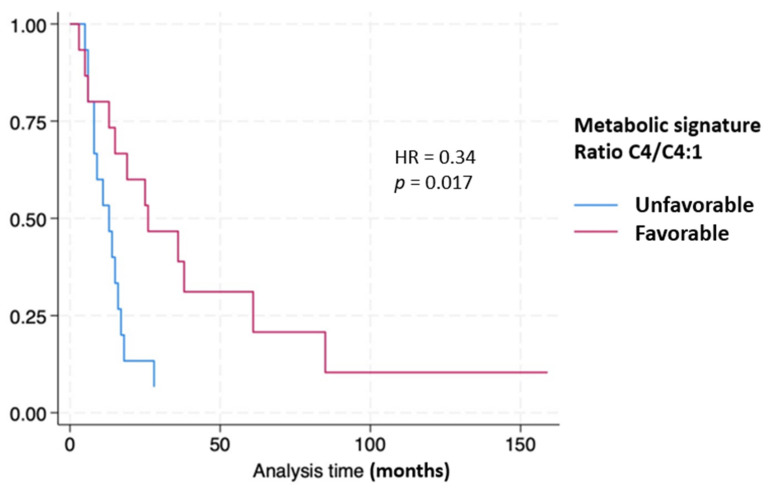
Kaplan–Meier curves stratifying pancreatic cancer patient survival based on metabolic signature.

An examination of PDAC patient overall survival divided at the median numeric value of the ratio C4/C4:1 of 6.87 by Kaplan–Meier plot revealed a hazard ratio of 0.34 (*p* = 0.017) dividing the favorable from the unfavorable groups (Figure 6).

### Comparison of Diagnostic and Prognostic Signatures

Several metabolic signatures were found to be diagnostic of PDAC. Among the most discriminating was the ratio [(C5-M-DC/PC ae C40:1)/C5:1-DC] with an area under the curve [AUC: 0.916 (0.838–0.977) *p* = 3.44 × 10^−14^] (Supplemental Figure S7).

An exploratory analysis examining the PDAC patients with the longest survival, i.e., greater than 36 months (n = 4), identified distinct prognostic signatures that were different from the diagnostic signatures. These include the ratios (Gly/PC ae C38:2) with an AUC = 1.0 (1:1) and (Putrescine/PC aa C32:0) AUC = 1.0 (1:1) (Figure 4A,B).

The ratio of C4/C4:1 divided at the median provided prognostic information as seen in Figure 6.

## 4. Discussion

We applied targeted metabolomics to a cohort of 10 patients with histologically confirmed pancreatic cancer and compared the results with those obtained from 169 healthy controls under an IRB-approved protocol. The exploratory analysis identified metabolic signatures that distinguished pancreatic cancers from the control groups.

We then expanded the analysis to a cohort of 30 PDAC patient plasmas and compared the results with 60 age- and sex-matched normal controls. The signatures proved highly discriminatory for the presence of PDAC.

To explore the prognostic implications of these metabolite signatures, we used the ratio of C4/C4:1 divided at the median value and compared the results with overall survival measured in months. The Kaplan–Meier curves reveal that patient survivals correlate strongly with the C4/C4:1 ratio with a hazard ratio (HR) of 0.34 (*p* = 0.017).

Additional metabolite ratios were explored that distinguished PDAC patient survivals. Among these were the ratios of the amino acid Glycine divided by the phosphatidyl choline ae 38:2 (Gly/PC ae 38:2) and the biogenic amine Putrescine divided by the phosphatidyl choline ae 32:0 (Putrescine/PC ae 32:0) (Figure 4A,B).

Among the findings is the marked diminution in the plasma levels of the branched-chain amino acids leucine and valine (Supplemental Figure S1A,B) as well as a reduction in the circulating levels of the structural lipids sphingomyelin and arachidonic lysophosphatidylcholine (Supplemental Figure S2A,B). The findings could reflect the well-described phenomenon of cancer cell nutrient hyper-consumption [26,27] or chronic malabsorption. Inflammatory bowel conditions resulting in malabsorption have previously been linked to colon, small bowel, lymphoma, cholangiocarcinoma [28] and pancreatic cancer [29].

To the contrary, plasma hexoses and dicarboxylic acyl-carnitines including methyl-glutaryl-carnitine (C5-M-DC) levels were markedly elevated in PDAC patients when compared with normal controls (Supplemental Figure S3A,B).

Elevated dicarboxylic acylcarnitines are associated with increased ῳ-oxidation of fatty acids, known to occur under conditions of starvation, diabetes and insulin resistance [30,31]. These conditions are associated with β-oxidation inhibition or deficiency and suggest mitochondrial dysfunction [32].

Another finding was low creatinine in the PDAC patients (Supplemental Figure S4). Prior studies have shown low creatinine levels to be an independent predictor of in-hospital mortality, suggesting liver dysfunction but when found in association with normal albumin levels; physical deconditioning or muscle wasting has been suggested as the cause [33].

Elevation of hexoses is characteristic of diabetes; however, unlike diabetic patients who have markedly elevated BCAAs [34], the PDAC patients showed elevated hexoses in the face of markedly diminished BCAAs (Supplemental Figure S5A–C). Indeed, the ratio of one of the BCAAs, valine, over total hexoses (valine/hexose) was so highly discriminating that this ratio alone identified PDAC in the plasma with an ROC = 0.85 (0.77–0.92, *p* = 9.97 × 10^−9^) (Supplemental Figure S6).

The PDAC signatures incorporate several metabolites, some of which are known to be associated with malignant transformation. Alterations in C4 and C5 reflect fatty acid oxidation defects like short-chain acyl-CoA dehydrognease deficiency (SCADD) [35]. In newborns, symptoms of SCADD include hypoglycemia, hypotonia, failure to thrive and seizures. More subtle deficiencies may remain asymptomatic throught adult life, but fatty oxidation changes have been associated with carcinogenesis [36].

Glycine and Serine are essential precursors for the synthesis of proteins, nucleic acids and lipids. Glycine also affects cellular antioxidant capacity. Alterations in Glycine may reflect changes associated with malignant transformation [37]. This has also been reported in pancreatic cancer [38]. The observed altered Glycine/lipid ratios may reflect these changes.

Polyamines accumulate in rapidly proliferating tissues and have been associated with cancer progression [39]. Over-expression of this pathway has been reported in pancreatic cancer [40]. Putrescine concentrations diverge from other polyamines under conditions of stress and electrolyte imbalance that may influence mitochondrial function [41]. The Putrescine/PC ratio may serve as a proxy for metabolic dysfunction in pancreatic cancer patients.

The finding that the diagnostic signatures differed from the prognostic signatures may reflect metabolic perturbations that differ more in magnitude than substance. Changes in C5 (valerylcarnitine) and C4 (butyrylcarnitine) both reflect altered changes in short-chain fatty acid oxidation, but the former was more prominent in the diagnostic ratio while the latter more prominent in the prognostic ratio. Similarly, long-chain fatty acid changes were observed in both the diagnostic (C40:1) and prognostic (C38:2) signatures, again suggesting commonalities in the metabolic changes observed in PDAC.

Among this study’s weaknesses is the exploratory nature of the analysis, as this is among the first attempts to apply plasma quantitative (targeted) mass spectrometry to pancreatic cancer diagnosis and prognosis. The training set data consisted of only 10 original cases, while the confirmatory data set includes only 30 individuals. We have made every effort to confirm the signatures including Monte Carlo and take-one-out methods, but biases in these small data sets cannot be entirely excluded. In addition, molecular profile results are incomplete, as many patients did not undergo next-generation sequence (NGS) testing. With NGS testing now more common, this should prove less of an issue going forward. Many of the findings reveal unanticipated metabolic pathway associations and remain hypothesis-generating, which will be the subject of further analysis as we accrue additional PDAC patients. Future studies will include more patients with non-malignant pancreatic conditions such as acute and chronic pancreatitis, pseudocysts and adenomas to further refine the performance characteristics of the identified signatures.

## 5. Conclusions

We conclude that pancreatic carcinogenesis, known to be associated with genomic aberrancies that include *K-RAS*, *TP53*, *CDKN2A/B*, *PIK3CA*, *SMAD-4*, DNA damage repair deficiency (i.e., *BRCA*, *ATM*) [42,43] and immune evasion, may also reflect altered metabolism as a mediator of malignant transformation. The pivotal role of the pancreas in human metabolism—sensing nutrient input, regulating critical digestive enzymes and responding to and regulating hexose, lipid and amino acid levels via insulin, glucagon and bioactive enteric hormones [44]—suggests that the study of metabolomics may offer the opportunity to unravel the complex origin of this highly lethal disease. By applying targeted NexGen metabolomics to interrogate the biochemical features of PDAC in patient plasma, we are working to develop diagnostic and prognostic tests to further improve the clinical management of PDAC.

## Data Availability

Data utilized in the preparation of this manuscript are reported in the manuscript and provided as Supplemental Materials (Supplemental Figures S1–S9, Tables S1–S3).

## Supplementary Materials

The following supporting information can be downloaded at: https://www.mdpi.com/article/10.3390/metabo14030148/s1, Table S1: Clinical characteristics of PDAC patients; Table S2: Clinical characteristics of controls; Table S3:The AbsoluteIDQ® p180 kit by Biocrates AG Life Sciences with all of the analytes and their standard nomenclature; Figure S1: A comparison of plasma BCAA (Valine (A) and Leucine (B) concentrations from pancreatic cancer patients (PanCA) and normal healthy controls (Controls); Figure S2: A comparison of plasma sphingomyelin (SM (OH) C22:1) (A) and Phosphatidyl choline (PC ae C40:1) (B) concentrations from pancreatic cancer patients (PanCA) and normal healthy controls (Controls); Figure S3: A comparison of plasma Hexoses (A) and methyl-glutaryl-carnitine (C5-M-DC) (B) concentrations from pancreatic cancer patients (PanCA) and normal healthy controls (Controls); Figure S4: A comparison of plasma creatinine concentrations from pancreatic cancer patients (PanCA) and normal healthy controls (Controls); Figure S5: A comparison of plasma BCAA (Valine (A) and Leucine (B) concentrations with hexose levels (C) from pancreatic cancer patients (PanCA) and normal healthy controls (Controls); Figure S6: Receiver Operator Curve (ROC) depicting the ratio of Valine (Val) to Hexose (H1) in plasma from patients with pancreatic cancer (PanCA) and normal healthy controls (Controls); Figure S7: Receiver Operator Curve (ROC) depicting the ratio of (C5-M-DC/PC ae C40:1) to C5:1-DC in plasma from patients with pancreatic cancer (PanCA) and normal healthy controls (Controls); Figure S8: PCA unsupervised analysis where pancreatic cancer (PDAC) samples were analyzed against other malignant conditions: liver cancer (30), lung cancer (23), colon cancer (85), head/neck cancer (58), hematologic cancer (65),breast cancer (58); and against samples from patients with non-malignant metabolic and immunological chronic conditions: latest ages of metabolic syndrome (70),HCV-induced cirrhosis (30); hyperthyroidism (8); hypothyroidism (8); HIVinfection (18); polycystic ovary syndrome (49); autoimmunedisease (86) and with control samples from healthy individuals (169); Figure S9: Pearson Moment (R-values) metabolite and ratios correlations with PDAC diagnosis.
