# Supplementary material for: Diagnostic and Prognostic Performance of Metabolic Signatures in Pancreatic Ductal Adenocarcinoma: The Clinical Application of Quantitative NextGen Mass Spectrometry
Source: Metabolites. 2024 Feb 29;14(3):148. doi: 10.3390/metabo14030148 (PMC10972340; doi:10.3390/metabo14030148)
Supplement: Supplementary file 1 [file metabolites-14-00148-s001.zip › Supplemental Figures.pdf]

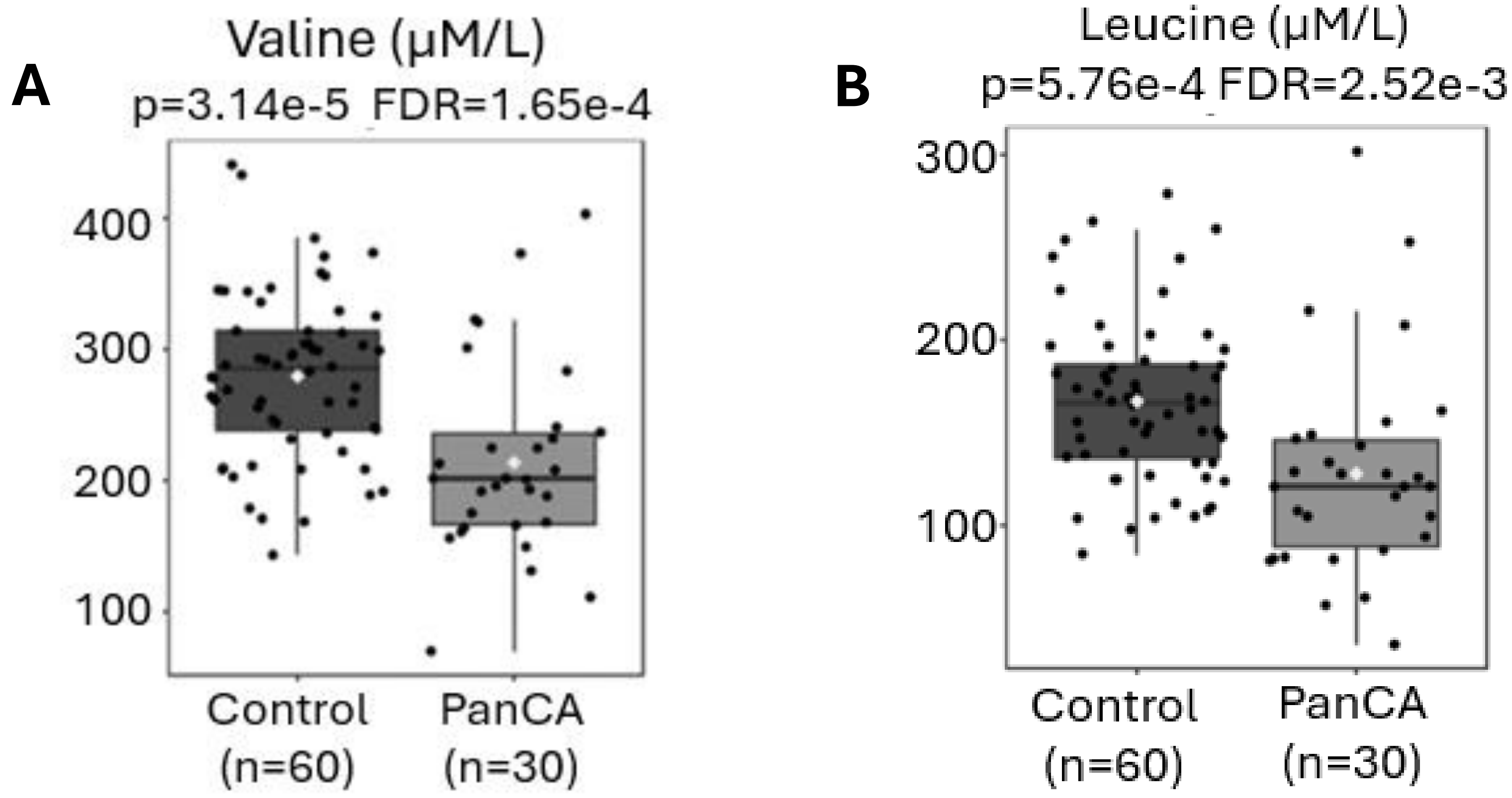

Supplemental Figure S1 (A and B) – A comparison of plasma BCAA (Valine (A) and Leucine (B) concentrations from pancreatic cancer patients (PanCA) and normal healthy controls (Controls)

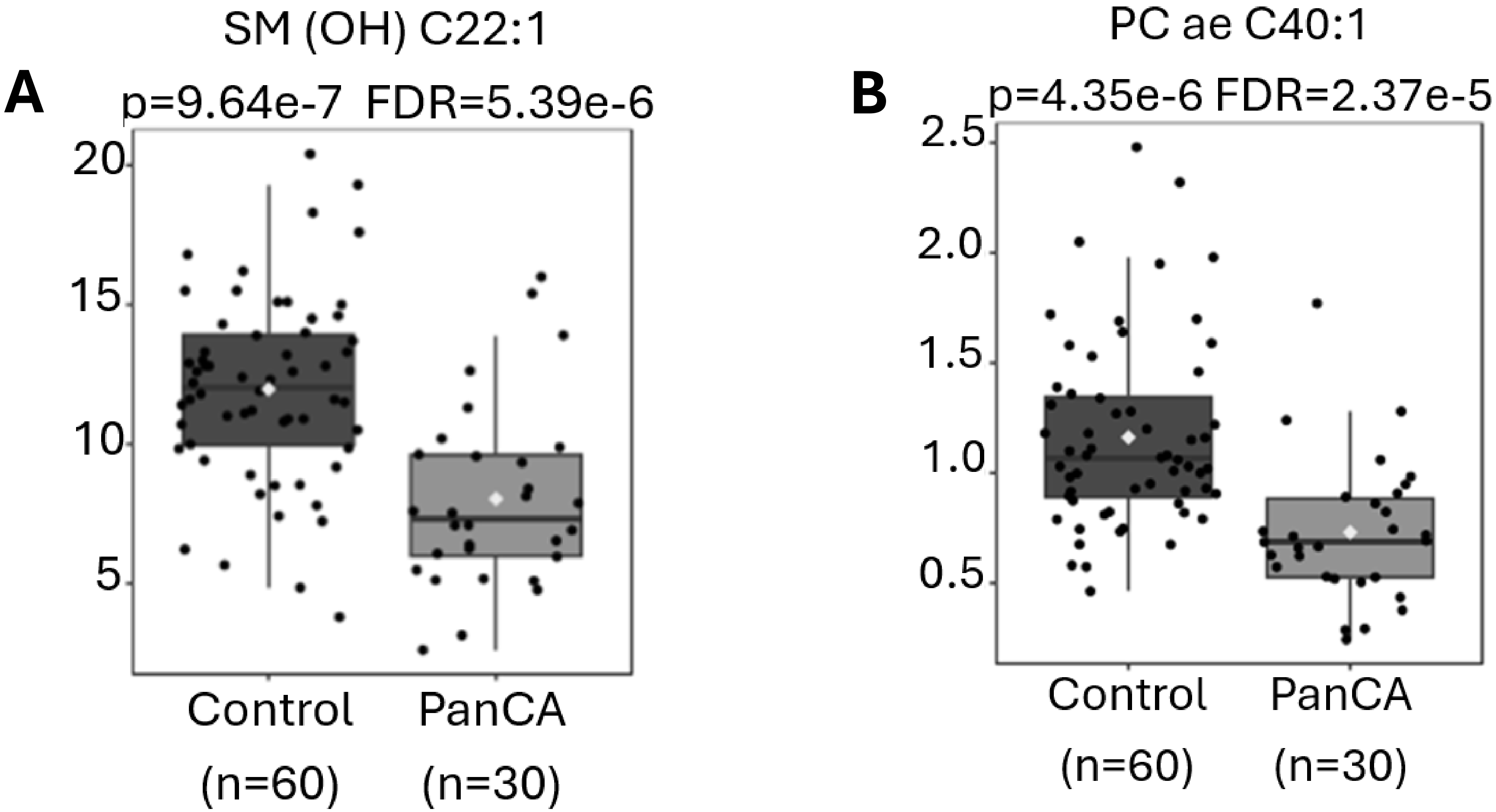

Supplemental Figure S2 (A and B) – A comparison of plasma sphingomyelin (SM (OH) C22:1) (A) and Phosphatidyl choline (PC ae C40:1) (B) concentrations from pancreatic cancer patients (PanCA) and normal healthy controls (Controls)

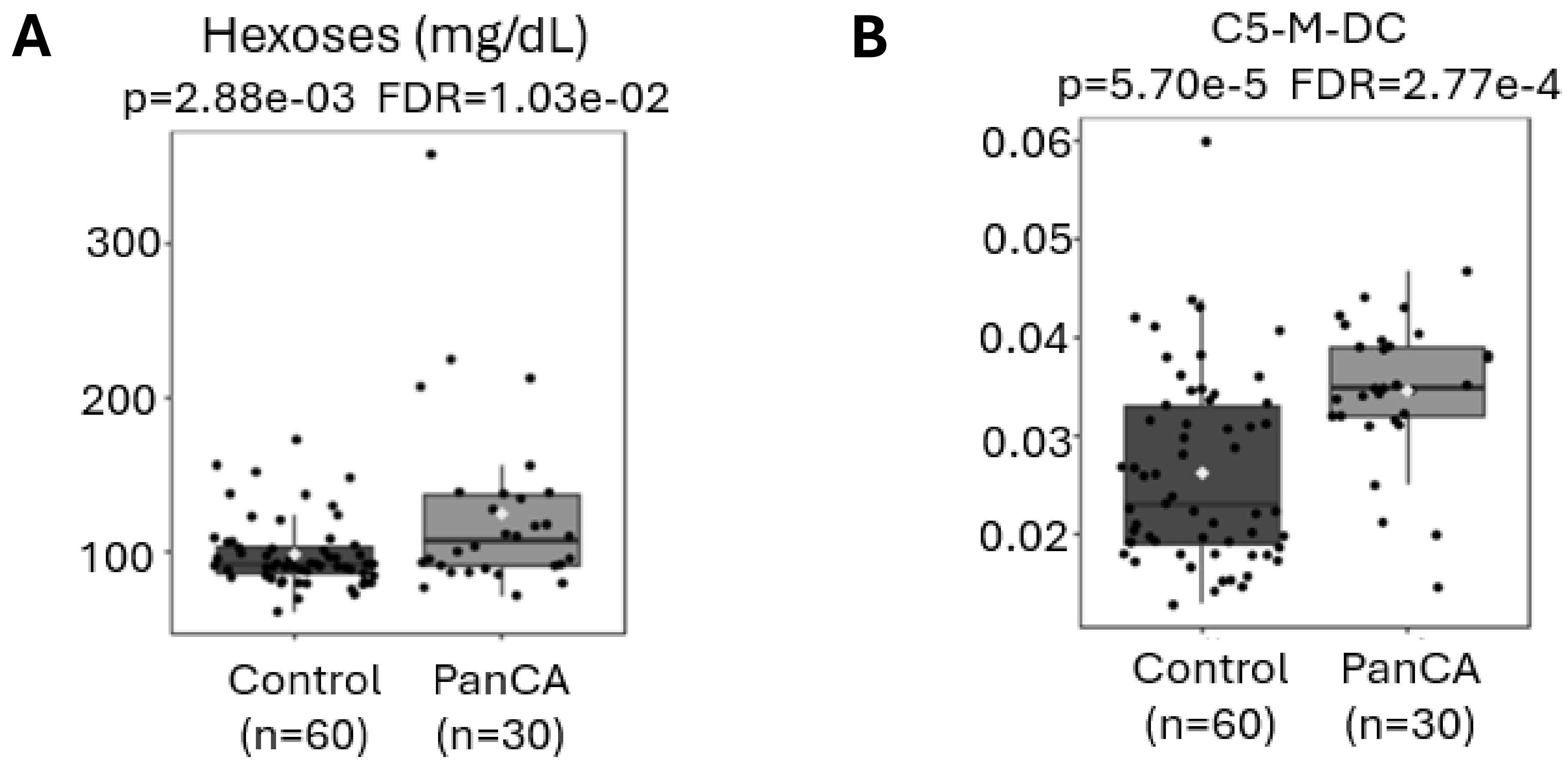

Supplemental Figure 3 (A and B): A comparison of plasma Hexoses (A) and methyl-glutaryl-carnitine (C5-M-DC) (B) concentrations from pancreatic cancer patients (PanCA) and normal healthy controls (Controls)

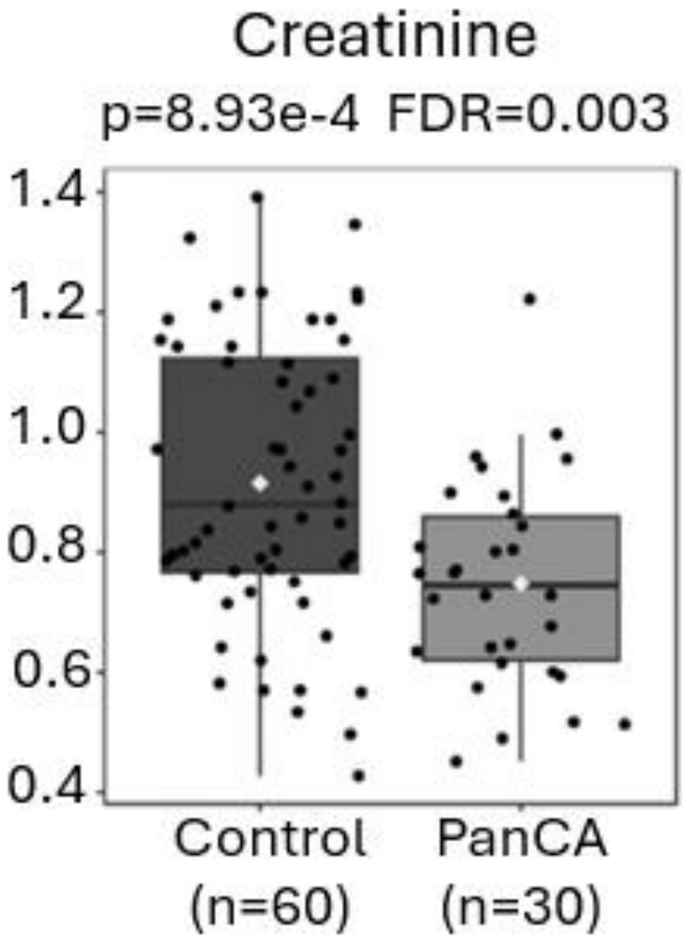

Supplemental Figure S4 – A comparison of plasma creatinine concentrations from pancreatic cancer patients (PanCA) and normal healthy controls (Controls).

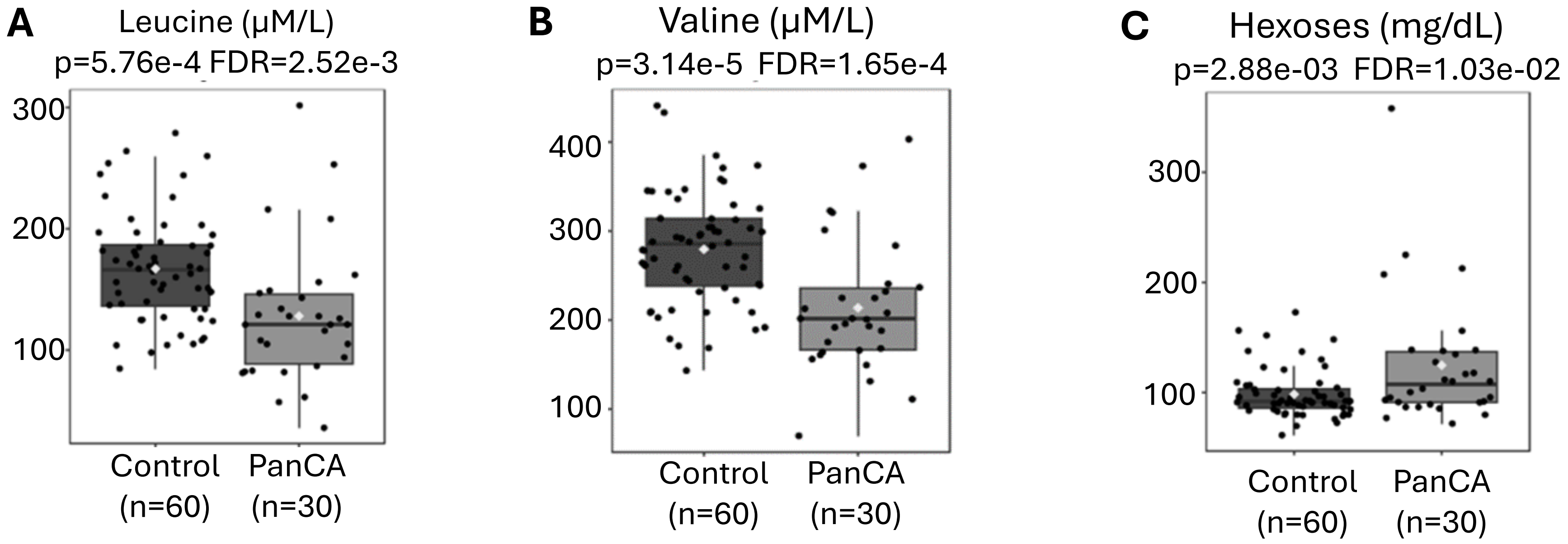

Supplemental Figure S5 (A, B and C) – A comparison of plasma BCAA (Valine (A) and Leucine (B) concentrations with hexose levels (C) from pancreatic cancer patients (PanCA) and normal healthy controls (Controls).

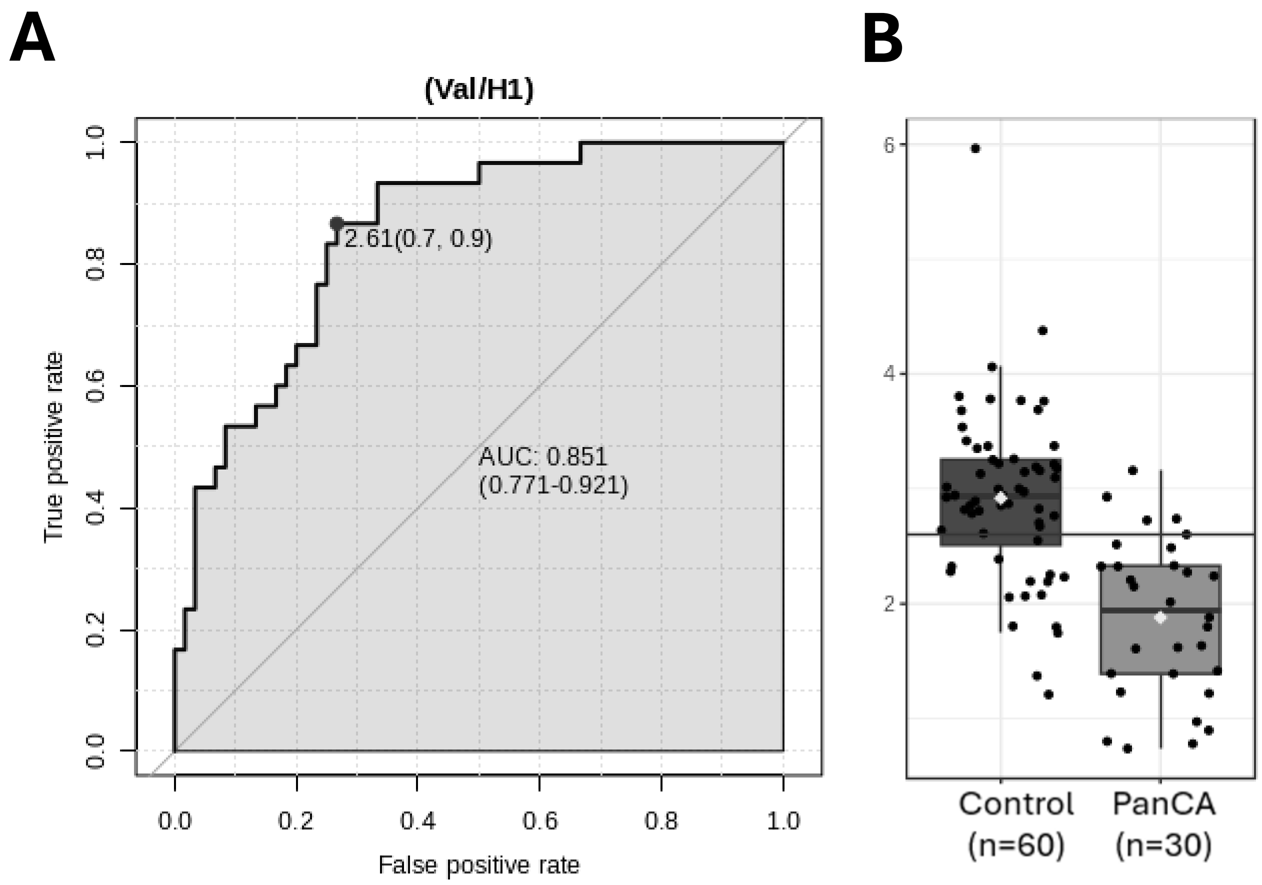

Supplemental Figure S6 – Receiver Operator Curve (ROC) depicting the ratio of Valine (Val) to Hexose (H1) in plasma from patients with pancreatic cancer (PanCA) and normal healthy controls (Controls).

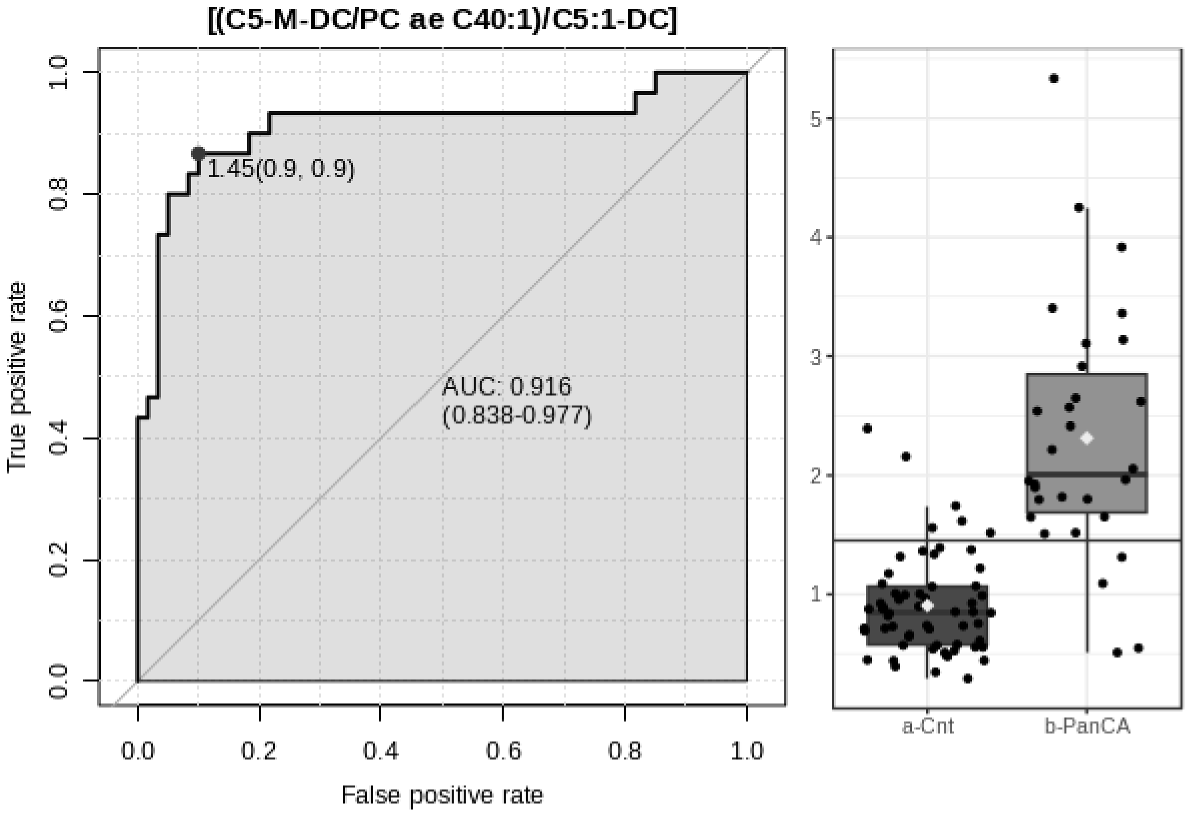

Supplemental Figure S7 – Receiver Operator Curve (ROC) depicting the ratio of (C5-M-DC/PC ae C40:1) to C5:1-DC in plasma from patients with pancreatic cancer (PanCA) and normal healthy controls (Controls).

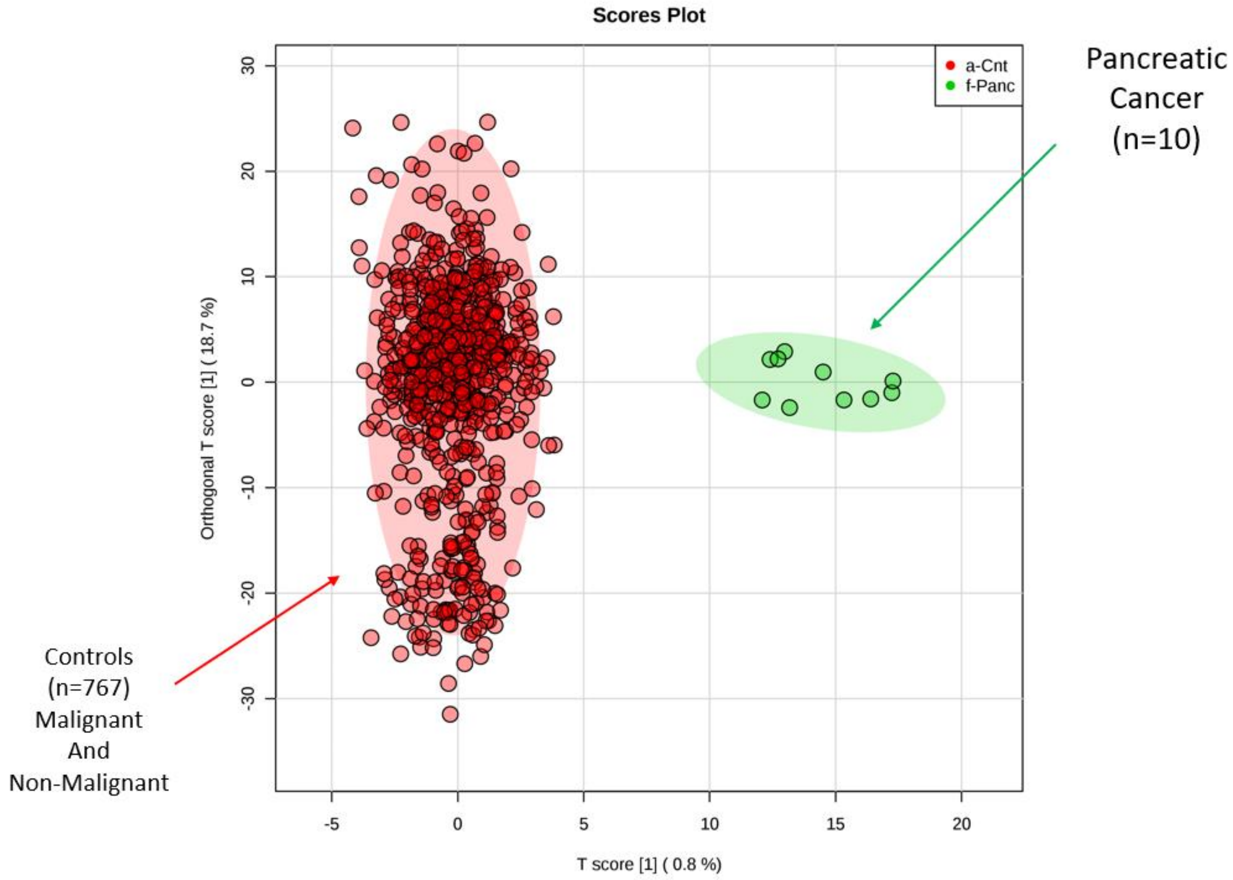

Supplemental Figure S8 – PCA unsupervised analysis where pancreatic cancer (PDAC) samples were analyzed against other malignant conditions: liver cancer (30), lung cancer (23), colon cancer (85), head/neck cancer (58), hematologic cancer (65), breast cancer (58); and against samples from patients with non-malignant metabolic and immunological chronic conditions: late stages of metabolic syn-drome (70), HCV-induced cirrhosis (30); hyperthyroidism (8); hypothyroidism (8); HIV infection (18); polycystic ovary syndrome (49); auto immune disease (86) and with control samples from healthy individuals (169).

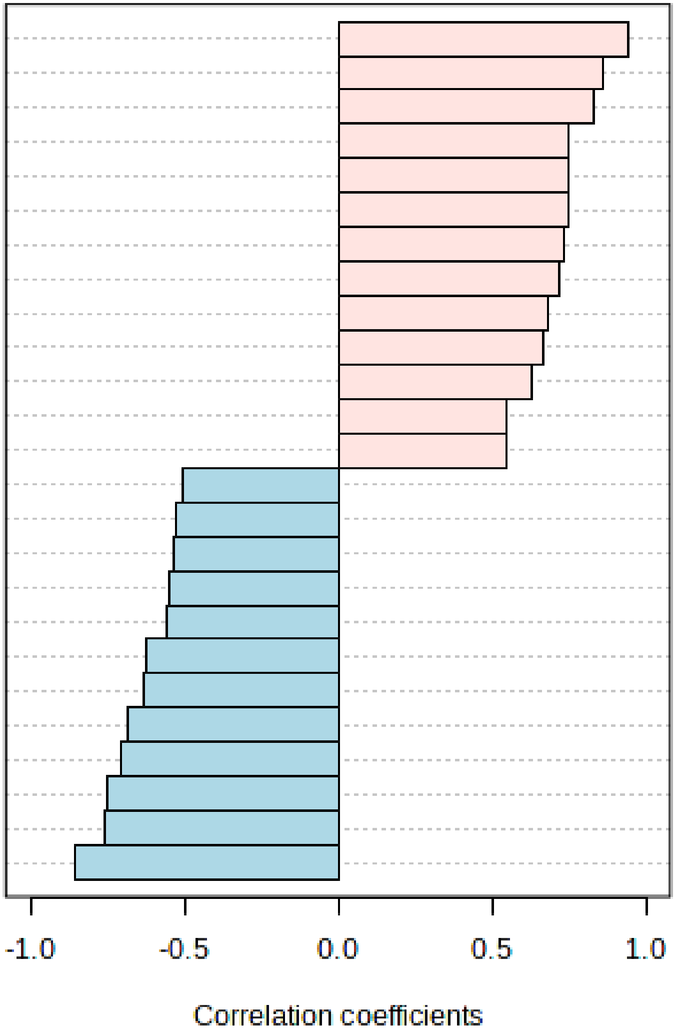

Ratio/Metabolites	Correlation	pValues	FDR
C9	0.95	7.1479e-89	7.8412e-86
C4	0.86	4.6776e-54	2.5657e-51
[(C12-DC/C16:2-OH)/(C9/C14:1-OH/C9/C16:2-OH)]	0.83	1.7528e-46	4.807e-44
[(C12-DC/C16:2-OH)/(C16:2-OH/C16:1-OH)]	0.75	2.5757e-33	3.532e-31
(C12-DC/C16:2-OH)/(C16:2-OH/C16:1-OH)	0.75	2.5757e-33	3.532e-31
(C18:1/C16:2-OH)	0.75	4.8178e-33	5.8723e-31
(C12-DC/C16:2-OH)	0.73	3.467e-31	3.8033e-29
(C5:1-DC/C16:2-OH)	0.72	1.9312e-29	1.926e-27
(C16-OH/C16:2-OH)(C16:2-OH/C16:1-OH)	0.68	4.7252e-26	3.9873e-24
(C5-DC/C16:2-OH)	0.66	3.443e-24	2.518e-22
(C16-OH/C16:2-OH)	0.63	1.8965e-21	1.3003e-19
(C9/C16:2-OH)	0.55	2.4505e-15	1.2219e-13
(C9/C16:2-OH)	0.55	2.4505e-15	1.2219e-13
Leu	-0.50	9.4626e-13	4.1522e-11
(SM OH C22:1/SM C18:0)/(PC ae C30:2/SM C24:0)/(C16:2-OH/C16:1-OH)	-0.53	2.6049e-14	1.1906e-12
PC ae C42:0	-0.54	1.0192e-14	4.861e-13
(Ser/C12-DC)	-0.55	1.817e-15	9.9663e-14
Asp	-0.56	4.3495e-16	2.5113e-14
(C162-OH/C16:1-OH)	-0.63	5.7569e-21	3.5085e-19
(AcylC-OH/Acyl-DC)	-0.63	2.16e-21	1.3939e-19
(C9/C14:1-OH)/C9/C16:2-OH)	-0.68	8.1212e-26	6.3635e-24
(C16:2-OH/C18:1-OH)/(PC aa C34:3/PC aa C34:4)	-0.71	2.0873e-28	1.9081e-26
C16:2-OH/C18:1-OH	-0.75	2.0232e-33	3.532e-31
[C16:2-OH/(PC aa C34:3/PC aa C34:4)]	-0.75	3.6689e-34	8.0495e-32
C16:2-OH	-0.85	9.4129e-52	3.442e-49

Supplemental Figure S9 – Pearson Moment (R-values) metabolite and ratios correlations with PDAC diagnosis.
