# Supplementary material for: Diagnostic and Prognostic Performance of Metabolic Signatures in Pancreatic Ductal Adenocarcinoma: The Clinical Application of Quantitative NextGen Mass Spectrometry
Source: Metabolites. 2024 Feb 29;14(3):148. doi: 10.3390/metabo14030148 (PMC10972340; doi:10.3390/metabo14030148)
Supplement: Supplementary file 1 [file metabolites-14-00148-s001.zip › Supplemental Table S3.pdf]

Supplemental Table S3 – The *AbsoluteIDQ® p180* kit by Biocrates AG Life Sciences with all of the analytes and their standard nomenclature.

Amino acids (21)			
Ala	Alanine	Lys	Lysine
Arg	Arginine	Met	Methionine
Asn	Asparagine	Orn	Ornithine
Asp	Aspartate	Phe	Phenylalanine
Cit	Citrulline	Pro	Proline
Glu	Glutamate	Ser	Serine
Gln	Glutamine	Thr	Threonine
Gly	Glycine	Trp	Tryptophan
His	Histidine	Tyr	Tyrosine
Ile	Isoleucine	Val	Valine
Leu	Leucine		

Biogenic amines (21)			
Ac-Orn	Acetylornithine	Met-SO	Methionine sulfoxide
alpha-AAA	alpha-Aminoadipic acid	Nitro-Tyr[1]	Nitrotyrosine
ADMA	Asymmetric dimethylarginine	PEA	Phenylethylamine
Carnosine	Carnosine	Putrescine	Putrescine
Creatinine	Creatinine	Sarcosine	Sarcosine
DOPA	Dihydroxyphenylalanine	Serotonin	Serotonin
Dopamine	Dopamine	Spermidine	Spermidine
Histamine	Histamine	Spermine	Spermine
c4-OH-Pro	*cis*-4-Hydroxyproline	SDMA	Symmetric dimethylarginine
t4-OH-Pro	*trans*-4-Hydroxyproline	Taurine	Taurine
Kynurenine	Kynurenine		

Acylcarnitines (40)			
C0	Carnitine	C5-DC (C6-OH)	Glutarylcarnitine (Hydroxyhexanoylcarnitine)
C2	Acetylcarnitine	C5-M-DC	Methylglutarylcarnitine
C3	Propionylcarnitine	C5-OH (C3-DC-M)	Hydroxyvalerylcarnitine (Methylmalonylcarnitine)
C3-OH	Hydroxypropionylcarnitine	C5:1	Tiglylcarnitine
C3:1	Propenoylcarnitine	C5:1-DC	Glutaconylcarnitine
C4	Butyrylcarnitine	C6 (C4:1-DC)	Hexanoylcarnitine (Fumarylcarnitine)
C4-OH (C3-DC)	Hydroxybutyrylcarnitine (Malonylcarnitine)	C6:1	Hexenoylcarnitine
C4:1	Butenylcarnitine	C7-DC	Pimeloylcarnitine
C5	Valerylcarnitine	C8	Octanoylcarnitine
C9	Nonaylcarnitine	C14:2-OH	Hydroxytetradecadienylcarnitine
C10	Decanoylcarnitine	C16	Hexadecanoylcarnitine
C10:1	Decenoylcarnitine	C16-OH	Hydroxyhexadecanoylcarnitine
C10:2	Decadienylcarnitine	C16:1	Hexadecenoylcarnitine
C12	Dodecanoylcarnitine	C16:1-OH	Hydroxyhexadecenoylcarnitine
C12-DC	Dodecanedioylcarnitine	C16:2	Hexadecadienylcarnitine
C12:1	Dodecenoylcarnitine	C16:2-OH	Hydroxyhexadecadienylcarnitine
C14	Tetradecanoylcarnitine	C18	Octadecanoylcarnitine
C14:1	Tetradecenoylcarnitine	C18:1	Octadecenoylcarnitine
C14:1-OH	Hydroxytetradecenoylcarnitine	C18:1-OH	Hydroxyoctadecenoylcarnitine
C14:2	Tetradecadienylcarnitine	C18:2	Octadecadienylcarnitine

Glycerophospholipids (90)			
lysoPC a C14:0	PC aa C34:1	PC aa C42:0	PC ae C38:2
lysoPC a C16:0	PC aa C34:2	PC aa C42:1	PC ae C38:3
lysoPC a C16:1	PC aa C34:3	PC aa C42:2	PC ae C38:4
lysoPC a C17:0	PC aa C34:4	PC aa C42:4	PC ae C38:5
lysoPC a C18:0	PC aa C36:0	PC aa C42:5	PC ae C38:6
lysoPC a C18:1	PC aa C36:1	PC aa C42:6	PC ae C40:1
lysoPC a C18:2	PC aa C36:2	PC ae C30:0	PC ae C40:2
lysoPC a C20:3	PC aa C36:3	PC ae C30:1	PC ae C40:3
lysoPC a C20:4	PC aa C36:4	PC ae C30:2	PC ae C40:4
lysoPC a C24:0	PC aa C36:5	PC ae C32:1	PC ae C40:5
lysoPC a C26:0	PC aa C36:6	PC ae C32:2	PC ae C40:6
lysoPC a C26:1	PC aa C38:0	PC ae C34:0	PC ae C42:0
lysoPC a C28:0	PC aa C38:1[2]	PC ae C34:1	PC ae C42:1
lysoPC a C28:1	PC aa C38:3	PC ae C34:2	PC ae C42:2
PC aa C24:0	PC aa C38:4	PC ae C34:3	PC ae C42:3
PC aa C26:0	PC aa C38:5	PC ae C36:0	PC ae C42:4
PC aa C28:1	PC aa C38:6	PC ae C36:1	PC ae C42:5
PC aa C30:0	PC aa C40:1	PC ae C36:2	PC ae C44:3
PC aa C30:2[2]	PC aa C40:2	PC ae C36:3	PC ae C44:4
PC aa C32:0	PC aa C40:3	PC ae C36:4	PC ae C44:5
PC aa C32:1	PC aa C40:4	PC ae C36:5	PC ae C44:6
PC aa C32:2	PC aa C40:5	PC ae C38:0	
PC aa C32:3	PC aa C40:6	PC ae C38:1	

Sphingomyelins (15)			
SM (OH) C14:1	SM C18:0	SM (OH) C22:2	SM (OH) C24:1
SM C16:0	SM C18:1	SM C22:3[2]	SM C26:0
SM C16:1	SM C20:2	SM C24:0	SM C26:1
SM (OH) C16:1	SM (OH) C22:1	SM C24:1	

Monosaccharides (1)			
H1	Hexoses (including glucose)		
